# When Iron Bites Back: Severe Thrombocytopenia After Intravenous Iron Sucrose Therapy (Venofer)

**DOI:** 10.7759/cureus.109185

**Published:** 2026-05-19

**Authors:** Muhammad Ali Lak, Aswathi Paleri, Manisha Satheesh, Dina Abdelhamid, Micaela Prisco, Uswah Shoaib, Abdullah Ahmad

**Affiliations:** 1 Internal Medicine, Icahn School of Medicine at Mount Sinai, Queens Hospital Center, New York City, USA; 2 Community Medicine, Combined Military Hospital (CMH) Lahore Medical College and Institute of Dentistry, Lahore, PAK; 3 Medicine, Hameed Latif Teaching Hospital, Lahore, PAK

**Keywords:** adverse drug reaction, drug-induced thrombocytopenia, intravenous iron therapy, iron deficiency anemia, iron sucrose, iron therapy complications, thrombocytopenia, venofer

## Abstract

Iron deficiency anemia (IDA) commonly presents with normal or elevated platelet counts, while thrombocytopenia is rare. Intravenous iron sucrose (Venofer) is widely used and generally well tolerated. We report the case of a 71-year-old female with chronic IDA who developed severe thrombocytopenia following iron sucrose therapy. Baseline hemoglobin was 6.3 g/dL with a platelet count of 229 × 10³/µL. After two doses of intravenous iron, hemoglobin improved to 9.3 g/dL, but platelets rapidly declined to 11 × 10³/µL. Extensive workup excluded thrombotic microangiopathy, autoimmune disease, infection, and malignancy, and peripheral smear showed no schistocytes. Iron therapy was discontinued, and supportive treatment, including platelet transfusions, led to gradual recovery. Naranjo scoring suggested a probable drug-induced reaction. This case highlights a rare but important adverse effect of intravenous iron therapy and underscores the need for close hematologic monitoring.

## Introduction

Iron deficiency anemia (IDA) is the most common cause of anemia worldwide in both adults and children, which is most often caused by gastroenterological and hematological causes [1]. IDA is often associated with mild thrombocytopenia; however, profound cases are rare. Venofer, iron sucrose, is a widely used and generally well-tolerated intravenous Food and Drug Administration (FDA)-approved medication for the treatment of IDA [2]. Intravenous use of iron is often the first-line regimen for people with poor absorption and those unable to tolerate oral forms. This case highlights the rare but severe presentation of iron-induced thrombocytopenia following intravenous iron therapy.

## Case presentation

A 71-year-old Caucasian female was evaluated for new-onset severe thrombocytopenia identified on routine laboratory testing during treatment for IDA. Her past medical history was significant for atrial fibrillation on apixaban for CHA₂DS₂-VASc-guided anticoagulation, prior pulmonary embolism on aspirin 81 mg, cerebrovascular accident with no residual deficits, schizophrenia managed with risperidone 2 mg nightly, hypertension on amlodipine 10 mg daily, hyperlipidemia on atorvastatin 40 mg nightly, and anemia. There was no documented history of alcohol use, substance abuse, or recent infections.

She was evaluated for severe thrombocytopenia found during routine labs secondary to the treatment for chronic microcytic anemia imposed upon IDA. Laboratory findings are presented in Table 1.

**Table 1:** Baseline laboratory investigations demonstrating severe iron deficiency anemia with normal leukocyte and platelet counts before the initiation of intravenous iron therapy.

Parameter	Lab result	Reference range
Hemoglobin	6.3 mg/dL	12.0–16.0 mg/dL
Platelet count	229 × 10³/µL	150–450 × 10³/µL
Leukocyte count	7.66 × 10³/µL	4.80–10.8 × 10³/µL
Iron	16 µg/dL	30–160 µg/dL
Iron saturation	7%	14–50%

She was asymptomatic, denying any shortness of breath, palpitation, dizziness, or chest pain. Physical examination was unremarkable except for pitting bilateral lower extremity edema with excoriations. She was scheduled for a colonoscopy, which revealed polyps, with pathology results consistent with tubular adenoma. As per Hematology and Oncology recommendations, the patient was scheduled for intravenous Venofer 200 mg for three separate days. Additional hematological, coagulation, and immunological investigations were performed to evaluate secondary causes of thrombocytopenia, all of which were unremarkable or non-contributory (Table 2).

**Table 2:** Serial complete blood count trends demonstrating hemoglobin improvement with progressive, severe thrombocytopenia following intravenous iron sucrose administration. WBC = white blood cell count; RBC = red blood cell count; HGB = hemoglobin; HCT = hematocrit; MCV = mean corpuscular volume; MCH = mean corpuscular hemoglobin; MCHC = mean corpuscular hemoglobin concentration; MPV = mean platelet volume; RDW = red cell distribution width; PLT = platelet count

Parameter	Ref range and units	8/17/2025	8/18/2025	8/19/2025	8/21/2025	8/22/2025	8/26/2025	8/28/2025	9/2/2025	9/10/2025
WBC	4.80–10.80 × 10³/µL	5.43	6.80	5.59	6.27	5.77	4.92	5.61	5.04	5.60
RBC	4.20–5.40 × 10⁶/µL	3.37	3.52	3.59	3.76	3.78	3.99	4.10	4.29	4.42
HGB	12.0–16.0 g/dL	8.8	9.2	9.9	9.9	10.4	10.7	11.2	10.0	11.6
HCT	37.0–47.0%	28.9	30.4	29.0	31.9	33.7	35.0	35.3	35.8	35.8
MCV	80.0–99.0 fL	85.8	84.7	86.6	84.8	84.4	85.4	82.3	81.0	81.0
MCH	27.0–31.0 pg	26.1	25.6	25.6	26.1	26.5	26.1	26.1	26.1	26.2
MCHC	29.8–35.2 g/dL	30.4	30.3	30.3	30.3	30.9	30.6	31.7	32.4	32.4
MPV	8.7–12.9 fL	-	-	-	-	-	-	-	-	-
RDW	12.0–15.0%	26.5	26.3	25.2	23.6	23.1	20.4	19.5	18.6	18.5
PLT	150–450 × 10³/µL	68	86	114	179	206	206	183	130	96
Neutrophil	44.0–70.0%	59.2	68.4	64.9	61.6	62.1	62.1	53.9	56.2	64.1

The patient had no contraindications to Venofer, specifically chronic kidney disease. After two doses of intravenous Venofer, her hemoglobin had increased to 9.3 mg/dL. However, the patient reported a significant decrease in platelet count: 75 × 10³/µL, 41 × 10³/µL, and 11 × 10³/µL subsequently. There was no evidence of active bleeding aside from vaginal spotting. Laboratory results showed a consistent drop in platelet count (Table 2).

As per the recommendations of Hematology and Oncology, Venofer was suspected as a potential cause of isolated thrombocytopenia and was discontinued after two intravenous doses, in addition to the discontinuation of aspirin and apixaban. A scheduled endoscopy was not performed due to thrombocytopenia. Further testing was performed to rule out the underlying cause of thrombocytopenia, which included but was not limited to systemic lupus erythematosus, paraproteinemia, and antiphospholipid antibody syndrome (Table 3).

**Table 3:** Additional hematological, coagulation, and immunological investigations performed to evaluate secondary causes of thrombocytopenia, with results largely within normal limits or non-contributory. ADAMTS13 = A disintegrin and metalloproteinase with thrombospondin type 1 motif member 13; RPR = rapid plasma reagin; TIBC = total iron-binding capacity; DRVTT = dilute Russell Viper venom time; APTT = activated partial thromboplastin time; IgG = immunoglobulin; AT III = antithrombin III; S/C = serum creatinine ratio; APL = anticardiolipin phospholipid

Parameter	Value	Unit	Reference range
ADAMTS13	137	%	50–150
Factor V	Normal	%	50–150
RPR titer	1:2	—	Non-reactive
Ferritin (6 months prior)	44	ng/mL	15–150
Protein C activity	Normal	%	70–140
AT III activity	107	%	80–120
Ferritin (current)	19	ng/mL	15–150
Protein S free antigen	66	%	60–130
Cardiolipin antibody IgA	7.3	APL U/mL	<20
TIBC	228	µg/dL	250–450
DRVVT screen	37.2	Seconds	30–45
Cardiolipin antibody IgG	<1.6	GPL U/mL	<20
Transferrin saturation	7	%	20–50
Silica clotting time	0.94	Ratio	0.8–1.2
APTT	37.2	Seconds	25–35
IgG kappa paraprotein	Weak	—	Absent
Beta-2 microglobulin	6.3	mg/L	0.7–1.8
Serum iron	16	µg/dL	50–170
S/C ratio	0.8	Ratio	0.7–1.3

The patient received oral ferrous sulfate 325 mg on an alternate-day dosing schedule. Hematology and Oncology services recommended platelet transfusion therapy, which the patient initially declined. Due to impaired decision-making capacity, the patient repeatedly refused multiple therapeutic interventions, including blood product transfusions and pelvic ultrasonography. Risperidone, prescribed for schizophrenia management, was discontinued given its rare association with thrombocytopenia. Infectious etiologies were considered unlikely contributors to the thrombocytopenia given the absence of leukocytosis, pyrexia, or systemic inflammatory symptoms.

Laboratory evaluation revealed vitamin D deficiency at 11.0 ng/mL (reference range: ≥20 ng/mL). Electrocardiography demonstrated QTc interval prolongation, measuring 444 milliseconds. CT of the head revealed no acute intracranial hemorrhage. Coagulation parameters showed activated partial thromboplastin time (aPTT) of 40.3 seconds, international normalized ratio (INR) of 1.3, and prothrombin time (PT) of 14.9 seconds. Following administration of two units of platelets and a single dose of prothrombin complex concentrate (K-Centra), repeat coagulation studies showed aPTT of 36.2 seconds and INR of 1.2.

Serial complete blood counts demonstrated progressive platelet recovery. Additional diagnostic workup, including hemoglobin electrophoresis, serum free light chains, and serum protein electrophoresis with immunofixation, yielded unremarkable results. Peripheral blood smear examination showed no schistocytes, effectively excluding thrombotic thrombocytopenic purpura and reducing suspicion for immune thrombocytopenic purpura. Ergocalciferol 50,000 IU weekly was initiated for vitamin D repletion. Serologic testing for syphilis returned positive by rapid plasma reagin, prompting initiation of doxycycline 100 mg twice daily for 14 days to treat latent syphilis infection.

Beta-2 microglobulin was elevated at 6.3 mg/L (reference range: 0.8-2.2 mg/L), raising initial concern for multiple myeloma; however, this diagnosis was effectively excluded based on the absence of supporting clinical and laboratory evidence.

Following discontinuation of intravenous iron sucrose (Venofer) and administration of serial platelet transfusions, platelet counts demonstrated sustained improvement, reaching 114 × 10³/µL. Hemoglobin levels similarly trended upward to 9.2 g/dL. The patient subsequently continued management of schizophrenia. Venofer was permanently discontinued due to its probable causative role in the severe thrombocytopenia. Oral iron supplementation was continued with plans for gradual hemoglobin normalization and ongoing outpatient hematologic surveillance.

Application of the Naranjo Adverse Drug Reaction Probability Scale yielded findings consistent with drug-induced severe thrombocytopenia secondary to Venofer. Key supporting evidence included temporal correlation between Venofer administration and thrombocytopenia onset, precipitous decline in platelet count, subsequent recovery following drug withdrawal, and systematic exclusion of alternative etiologies. The clinical course aligned with typical drug-induced immune thrombocytopenia patterns, with platelet recovery occurring within days to weeks after cessation of the offending agent.

## Discussion

In developed countries, IDA most commonly results from chronic blood loss or impaired iron absorption. Malabsorption may occur in conditions such as atrophic gastritis and celiac disease, as well as following gastrointestinal surgeries [1]. Chronic blood loss from gastrointestinal, genitourinary, or gynecological sources remains a common etiology of IDA, with excessive menstruation being the leading cause in premenopausal women [3].

Hemodynamically stable patients with IDA resulting from chronic blood loss are typically prescribed oral iron therapy [4]. Available formulations include ferrous and ferric salts, with the ferrous form being more commonly used due to its superior absorption. Commonly used iron formulations include ferrous sulfate, ferrous fumarate, and ferrous gluconate, which differ in their elemental iron content [5]. The Centers for Disease Control and Prevention (CDC) recommends a daily elemental iron dose of 150-180 mg, administered in divided doses two to three times per day [5]. An increase in reticulocyte count is typically observed within the first week of iron therapy, while hemoglobin levels rise more gradually over the following one to two weeks. Gastrointestinal side effects, including nausea, vomiting, abdominal discomfort, constipation, and dark-colored stools, may limit adherence to oral iron therapy. To improve tolerability, enteric-coated and delayed-release formulations have been developed; however, these are associated with reduced absorption compared to non-enteric-coated preparations [6].

Intravenously administered iron is another therapeutic modality used for iron repletion in patients with blood loss exceeding 10 mL/day (about 5 mg iron). With intravenous iron therapy, desired serum iron levels can be achieved, and marrow erythropoietic activity may increase by fourfold to eightfold [7]. In the past, iron dextran was the most used formulation, which has since been replaced by newer, safer iron preparations [4]. Available intravenous iron preparations in the United States include iron dextran (INFeD® or DexFerrum®), iron sucrose (Venofer®), sodium ferric gluconate (Ferrlecit®), and ferumoxytol (Feraheme®) [4]. The most commonly reported adverse outcomes are mild, such as dysgeusia, hypotension, hypertension, nausea, and injection-site reaction [2]. Other reactions, such as hypersensitivity reactions, are noted. However, according to CDC records and drug labels, thrombocytopenia is not listed among the categories of side effects of iron formulations and has not been reported frequently [4]. The prevalence, mechanisms, and clinical significance of thrombocytopenia following iron repletion therapy in patients with IDA therefore remain a grey area that warrants further investigation [8].

Studies reveal iron deficiency is common in non-dialysis chronic kidney disease (ND-CKD) patients and requires parenteral iron therapy [8]. A retrospective analysis of ND-CKD patients with IDA treated with low-molecular-weight iron dextran (LMWID) between 2005 and 2009 showed that a decrease in platelet counts in response to iron replacement therapy was statistically significant (305.72 ± 108.86 vs. 255.58 ± 78.97, p < 0.0001) [8]. Concomitant use of darbepoetin did not significantly affect the observed decline in platelet counts [8].

Kulnigg-Dabsch et al. reported a retrospective analysis that evaluated the effect of iron therapy on platelet counts in patients with inflammatory bowel disease (IBD)-associated anemia. Records of 308 patients demonstrated a dose-dependent reduction in platelet counts (mean 425 G/L to 320 G/L; p < 0.001), independent of iron formulation (iron sulfate, iron sucrose, or ferric carboxy maltose). No association was observed with concomitant erythropoietin use or inflammatory markers, including leukocyte counts and C-reactive protein. Similar findings were reported in the FERGIcor cohort (n = 448), where platelet counts decreased from 383 G/L to 310 G/L (p < 0.001) [9].

Furthermore, thrombocytosis has been recognized as a marker of inflammation in patients with IBD. The ThromboVIT trial, a single-blinded, placebo-controlled, randomized study, evaluated the effect of ferric carboxy maltose (FCM) in IBD patients with secondary thrombocytosis (platelets > 450 G/L) [10]. A total of 26 patients were enrolled, with 15 included in the per-protocol analysis. A reduction in platelet count greater than 25% (primary endpoint) was observed in four of eight patients (50%) in the iron group compared to one of seven patients (14%) in the placebo group (p = 0.143). Additionally, mean platelet counts decreased significantly with FCM (536 G/L to 411 G/L) but not with placebo (580 G/L to 559 G/L; p = 0.002) [10].

Notably, Venofer iron sucrose is a widely used and generally well-tolerated FDA-approved intravenous medication for the treatment of IDA. However, a pediatric series of IDA reported that thrombocytopenia was commonly observed and consistently improved following Venofer therapy [11]. This contradicts our patient presentation, in which further intravenous iron supplementation was associated with a severe decrease in platelet count. A focused review noted that most patients reported low hemoglobin levels and variable platelet counts experienced a significant decrease in platelets after starting iron, with most of the cases self-correcting [4]. The parenteral route is more commonly associated with thrombocytopenia as an adverse effect, with discontinuation of iron therapy needed in two patients [4]. This was further strengthened by De Sanctis et al., who reported that in those with IDA receiving intravenous iron, parenteral iron was associated with cytopenia, specifically leukopenia and neutropenia [12].

These studies support that iron replacement therapy can lead to rare cases of cytopenias or specifically severe thrombocytopenia. Our patient’s course closely parallels such patterns by reporting IDA, intravenous iron supplementation of Venofer, onset of severe thrombocytopenia several days post-treatment, and subsequent recovery of platelet count following discontinuation and supportive care. This case adds to a very limited body of evidence that suggests iron-induced severe thrombocytopenia in patients without chronic kidney disease or other precipitating causes.

## Conclusions

This case highlights a rare but clinically significant adverse effect of intravenous iron therapy. A clear temporal association between iron sucrose administration and platelet decline, along with recovery following drug withdrawal and exclusion of alternative causes, supports a probable drug-induced mechanism. Clinicians should maintain awareness of this potential complication and consider close hematologic monitoring in patients receiving parenteral iron. Further studies are needed to better define the underlying mechanisms, incidence, and clinical implications of iron therapy-associated thrombocytopenia.
